# Structure-Based Virtual Screening and Identification of Potential Inhibitors of SARS-CoV-2 S-RBD and ACE2 Interaction

**DOI:** 10.3389/fchem.2021.740702

**Published:** 2021-09-27

**Authors:** Jiacheng Xiong, Yusen Xiang, Ziming Huang, Xiaohong Liu, Mengge Wang, Guangbo Ge, Hongzhuan Chen, Jianrong Xu, Mingyue Zheng, Lili Chen

**Affiliations:** ^1^ State Key Laboratory of Drug Research, Drug Discovery and Design Center, Shanghai Institute of Materia Medica, Chinese Academy of Sciences, Shanghai, China; ^2^ College of Pharmacy, University of Chinese Academy of Sciences, Beijing, China; ^3^ Institute of Interdisciplinary Integrative Medicine Research, Shanghai University of Traditional Chinese Medicine, Shanghai, China; ^4^ Shanghai Institute for Advanced Immunochemical Studies, School of Life Science and Technology, ShanghaiTech University, Shanghai, China; ^5^ Academy of Integrative Medicine, Shanghai University of Traditional Chinese Medicine, Shanghai, China; ^6^ Department of Pharmacology and Chemical Biology, Shanghai Jiao Tong University School of Medicine, Shanghai, China

**Keywords:** SARS-CoV-2, angiotensin-converting enzyme 2 (ACE2), spike protein receptor-binding domain (S-RBD), structure-based virtual screening, protein-protein interaction (PPI) inhibitors

## Abstract

The emergence and rapid spread of SARS-CoV-2 have caused a worldwide public health crisis. Designing small molecule inhibitors targeting SARS-CoV-2 S-RBD/ACE2 interaction is considered as a potential strategy for the prevention and treatment of SARS-CoV-2. But to date, only a few compounds have been reported as SARS-CoV-2 S-RBD/ACE2 interaction inhibitors. In this study, we described the virtual screening and experimental validation of two novel inhibitors (DC-RA016 and DC-RA052) against SARS-CoV-2 S-RBD/ACE2 interaction. The NanoBiT assays and surface plasmon resonance (SPR) assays demonstrated their capabilities of blocking SARS-CoV-2 S-RBD/ACE2 interaction and directly binding to both S-RBD and ACE2. Moreover, the pseudovirus assay revealed that these two compounds possessed significant antiviral activity (about 50% inhibition rate at maximum non-cytotoxic concentration). These results indicate that the compounds DC-RA016 and DC-RA052 are promising inhibitors against SARS-CoV-2 S-RBD/ACE2 interaction and deserve to be further developed.

## Introduction

There is an ongoing pandemic of coronavirus disease 2019 (COVID-19) caused by severe acute respiratory syndrome coronavirus 2 (SARS-CoV-2). SARS-CoV-2 is a kind of positive single-stranded RNA virus with an envelope structure (Gorbalenya et al., 2020; Yao et al., 2020). It was the seventh known coronavirus able to infect humans (Chen et al., 2020). The human infection caused by SARS-CoV-2 can induce severe pulmonary disease and complications with significant morbidities and mortalities (Sun et al., 2020; Yang X. et al., 2020; Zhang J.-j. et al., 2020). According to the released statistics from the World Health Organization (WHO), the numbers of confirmed cases and deaths of COVID-19 worldwide have so far exceeded 180 million and four million with a continuous upward tendency (https://covid19.who.int/table). Despite the disastrous effect of COVID-19 on public health, civil society, and the global economy, there is currently still no specific drug available against it. Hence, the search for effective treatment strategies for SARS-CoV-2 infections is in urgent demand.

**FIGURE 1:**
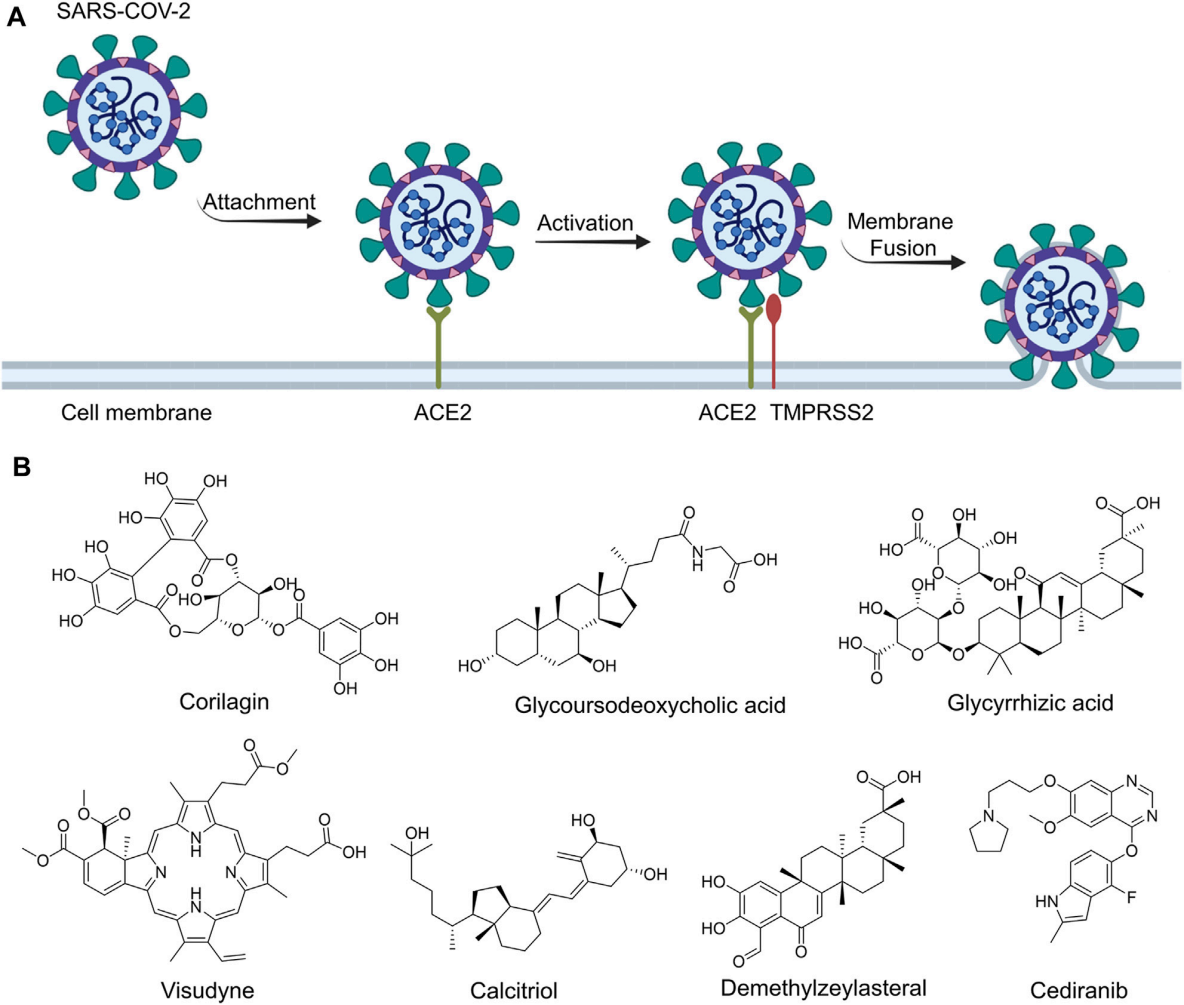
The routes of SARS-CoV-2 invade into host cells **(A)** and several representative inhibitors against SARS-CoV-2 S-RBD/ACE2 interaction **(B)**.

The invasion of SARS-CoV-2 into host cells relies on the spike protein on the surface of its envelope (Walls et al., 2020). In humans, the primary receptor of the SARS-CoV-2 spike protein is angiotensin-converting enzyme 2 (ACE2) (Wang et al., 2020; Zhang H. et al., 2020). SARS-CoV-2 spike protein recognizes and binds to ACE2 through the receptor-binding domain (Xu et al., 2021). Then it is hydrolytically activated by transmembrane protease serine 2 (TMPRSS2) and mediates subsequent virus-host cell membrane fusion (Figure 1A) (Hoffmann et al., 2020). The binding affinity between spike protein of SARS-CoV-2 and ACE2 has been determined to be low to ∼15 nM and 10 to 20 fold higher than that reported in SARS-CoV in 2002, which may be an important cause of the extremely high transmissibility of SARS-CoV-2 (Wrapp et al., 2020). Considering such a critical role of the interaction between spike protein receptor-binding domain (S-RBD) and ACE2 in the entry of the SARS-CoV-2 into host cells, the inhibition of such interaction is considered as a particularly attractive strategy for the development of treatments for SARS-CoV-2 infections (Li et al., 2020; Monteil et al., 2020; Yang J. et al., 2020).

Recently, virtual screening and high throughput screening studies targeting SARS-CoV-2 S-RBD/ACE2 interaction have discovered several small molecule inhibitors (Figure 1B) (Carino et al., 2020; Fu et al., 2020; Hanson et al., 2020; Yu et al., 2020; Zhu et al., 2021). Nevertheless, the antiviral activities of some compounds, such as corilagin, glycoursodeoxycholic acid, and glycyrrhizic acid, are not clear (Carino et al., 2020; Hanson et al., 2020; Yu et al., 2020). Visudyne shows intense antiviral activity against SARS-CoV-2 pseudovirus. However, its cytotoxicity data is not reported (Fu et al., 2020). Besides, demethylzeylasteral and cediranib have undergone pseudovirus and cytotoxicity tests, but only demethylzeylasteral shows slight inhibitory activity (approximately 7%) against SARS-CoV-2 pseudovirus under nontoxic concentration (Zhu et al., 2021). Hence, there remains a critical need and challenge to discover safe and effective inhibitors against the interaction between S-RBD and ACE2.

In this study, we identified two novel inhibitors (named DC-RA016 and DC-RA052) against SARS-CoV-2 S-RBD/ACE2 interaction through structure-based virtual screening and biological experiments. NanoLuc binary technology (NanoBiT)-based binding and surface plasmon resonance (SPR) assay demonstrated that DC-RA016 and DC-RA052 could bind to both S-RBD and ACE2, blocking the interaction between them. Additionally, the pseudovirus assay and cytotoxicity experiment showed that both DC-RA016 and DC-RA052 have moderate inhibition ability to SARS-CoV-2-S pseudovirus and low cytotoxicity. In addition, the preliminary pharmacophore analysis and the mechanism action study of DC-RA016 were carried out to further uncover the inhibitory mechanism of the compound on SARS-CoV-2 S-RBD/ACE2 interaction. Therefore, these two compounds are promising new inhibitors of SARS-CoV-2 S-RBD/ACE2 interaction and worth further development.

## Materials and Methods

### Structure-based Virtual Screening and 2D Similarity Search

The whole virtual screening process was carried out using Schrödinger Suite 2017 on a Linux server with four 6-core Intel Xeon E5-4607 CPUs and 32 GB of memory. The crystal structure of the SARS-CoV-2 S-RBD bound to ACE2 (PDB code: 6M0J) was obtained from the Protein Data Bank (PDB) for the docking studies. This crystal structure was prepared using the Protein Preparation Wizard module with default parameters. The process of protein preparation included the determination of valence bonds, the addition of hydrogen atom, removal of water molecule and heteroatom, optimization of hydrogen bond network, optimization of the orientation of Asn, Gln, and His, and restrained structural optimization. Finally, a grid box containing the contact interface of SARS-CoV-2 S-RBD/ACE2 complexes was generated with the Receptor Grid Generation module by manually setting the central coordinate. The size of the grid box was defined as 30 × 30 × 30 Å.

Small molecules in the SPECS commercial compound database were selected for virtual screening. In order to avoid false positives caused by pan assay interference compounds (PAINS), the compounds containing PAINS structural alert were removed with the Structure Filter module in Canvas. Subsequently, the three-dimensional coordinates, different stereoisomers, and protonation states at pH 7.0 ± 2.0 of remaining compounds were generated with LigPred Module. The resulting structures were used for docking.

The final molecular docking was performed with the Virtual Screening Workflow module. All treated molecules were first docked into the defined interface pocket in standard precision mode. Then the top poses were further docked in extra precision mode.

The structural similarity between the two molecules was calculated using the 1024-dimensional Morgan fingerprints with radius two and the Tanimoto coefficient. The calculation of fingerprints and the Tanimoto coefficient were both implemented with the RDkit python package.

### Compounds

The tested compounds were purchased from SPECS (Zoetermeer, Netherlands) and used directly without further purification. All compounds were first dissolved in DMSO and subsequently diluted to the final bioassay concentration.

### Determination of SARS-CoV-2 S-RBD/ACE2 Inhibitors by NanoBiT-Based Assay

The initial screening and 50% inhibitory concentration (IC_50_) determination of SARS-CoV-2 S-RBD/ACE2 interaction inhibitors were conducted as previously described (Yu et al., 2020). Briefly, SARS-CoV-2 S-RBD-LgBiT (S residues 319–591) and SmBiT-ACE2 (ACE2 residues 19–615) fusion plasmids were transiently co-transfected into HEK293 cells in a 6-well plate using FuGENE HD transfection reagent (Promega, Madison, WI). After 6 h, the HEK293 cells were reseeded into a 384-well plate for screening the active compounds. The compounds were added at indicated concentrations and plates were incubated for 3 h. After the addition of Nano-Glo live Cell Assay reagent, luminescence was determined using the Envision plate reader (EnVision, Perkin Elmer, Waltham, MA, United States). To exclude the false positives, the cytotoxicity of the compounds on the HEK293 cells and the inhibitory effects of the compounds on NanoLuc (HEK293/Nanoluc stable cells) were also measured, respectively. The activities of the compounds were evaluated using the inhibitory effects on SARS-CoV-2 S-RBD/ACE2 interaction (NanoBiT inh%), NanoLuc luciferase (NanoLuc inh%) and the cell proliferation (Cytotox inh%, CC_50_) on HEK293 cells.

### SARS-CoV-2-S Pseudovirus-Based Inhibition Assay

Three separate plasmids including pAX2, pHB-Rluc and pcDNA3.1-SARS-CoV-2-S were obtained from Precedo (Anhui, China). HEK293T cells were grown in Dulbecco’s modified Eagle’s medium (DMEM) (Corning Inc., Corning, NY, United States) supplemented with 10% heat-inactivated fetal bovine serum (FBS) (ExCell Bio, Shanghai, China) and 1% penicillin-streptomycin under a humidified atmosphere containing 5% CO_2_ at 37°C. HEK293T cells grown to 70% confluency were co-transfected with the above-mentioned plasmids using LipoFiter 3.0 transfection reagent (Hanbio, Shanghai, China) according to the manufacturer’s instruction. After 6 h of transfection, HEK293T cells were refreshed with DMEM containing 10% FBS and SARS-CoV-2-S pseudoviruses in the supernatant was harvested at 48 h, filtered using a 0.45 μm membrane (Jet Bio-Filtration, Guangzhou, China) and stored in aliquots at −80°C until use. Subsequently, HEK293T cells were transiently transfected with pcDNA3.1-ACE2 (Precedo, Anhui, China) or with vector alone. Transfected HEK293T cells were incubated with the indicated concentrations of test compounds at 37°C for 1 h. Then, SARS-CoV-2-S pseudovirus and Polybrene (6 μg/ml) (Absin, Shanghai, China) were added to infect the cells for 24 h. After that, the cells were further cultured with fresh DMEM containing 10% FBS for an additional 24 h. Then cells were lysed with Cell Lysis Buffer (Promega, Madison, WI, United States), and the luciferase activity was detected using a Multilabel Reader (SpectraMax Paradigm, Molecular Devices, CA, United States). The inhibition rate (%) was calculated by the equation: (the luminescence of the test compounds/the maximum luminescence after transfection for SARS-CoV-2-S pseudovirus) × 100%. IC_50_ values were determined via nonlinear regression analysis using GrapPad Prism software 8.0 (GraphPad Software, Inc., San Diego, CA, United States). The cytotoxicity of the test compounds was determined using the CellTiter-Glo (CTG) Luminescent Cell Viability Assay (Promega, Madison, WI, United States) according to the manufacturer’s protocols. Selectivity index (SI) for each compound was calculated by dividing CC_50_ (cytotoxicity on HEK293T cells) by IC_50_ (the inhibitory activity against SARS-CoV-2-S pseudovirus). The maximum non-cytotoxic concentration (MNCC) was defined as the concentration required to retain cell viability by 90% and calculated according to the previously published method (Cheng et al., 2013). The inhibition of the compounds to SARS-CoV-2-S pseudovirus infectivity at MNCC was fitted based on the inhibition curve and expressed as the inhibition ratio (%I).

### SPR–Based Assay

A BIAcore T200 instrument (GE Healthcare Life Sciences, United States) was used to evaluate the binding affinity of the test compounds to human ACE2 or SARS-CoV-2 S-RBD as previously described (Li et al., 2017). Briefly, both proteins were respectively immobilized on the different channels of CM5 sensor chip by a standard amine-coupling approach at a flow rate of 10 μL/min in 10 mM sodium acetate buffer (pH 4.0). The sensor surface was activated with a 7 min injection of the mixture of 50 mM N-hydroxysuccinimide (NHS) and 200 mM 1-ethyl-3-(3-dimethylaminopropyl) carbodiimide (EDC). Then 10 μg/ml of human ACE2 or 50 μg/ml of SARS-CoV-2 S-RBD was injected for 420 s and the surface was blocked with 1 M ethanolamine, pH 8.5. Series concentrations of the test compounds were injected into the flow system at a flow rate of 30 μL/min for 90 s, and the dissociation was 120 s. All binding analysis was performed in phosphate buffered saline (PBS) with 0.05% (v/v) Tween-20 and 1% DMSO (pH 7.4) at 25°C. Prior to analysis, double reference subtractions and solvent corrections were made to eliminate bulk refractive index changes, injection noise, and data drift. The binding affinity was determined by fitting to a Langmuir 1:1 binding model within the BIAcore Evaluation software (GE Healthcare Life Sciences, United States).

### Statistical Analysis

Statistical analyses were performed using GrapPad Prism software 8.0. One-way ANOVA was used to determine the statistical significance between different groups (*p < 0.05, **p < 0.01, and ***p < 0.001).

## Results and Discussion

### Structure-based Virtual Screening

**FIGURE 2:**
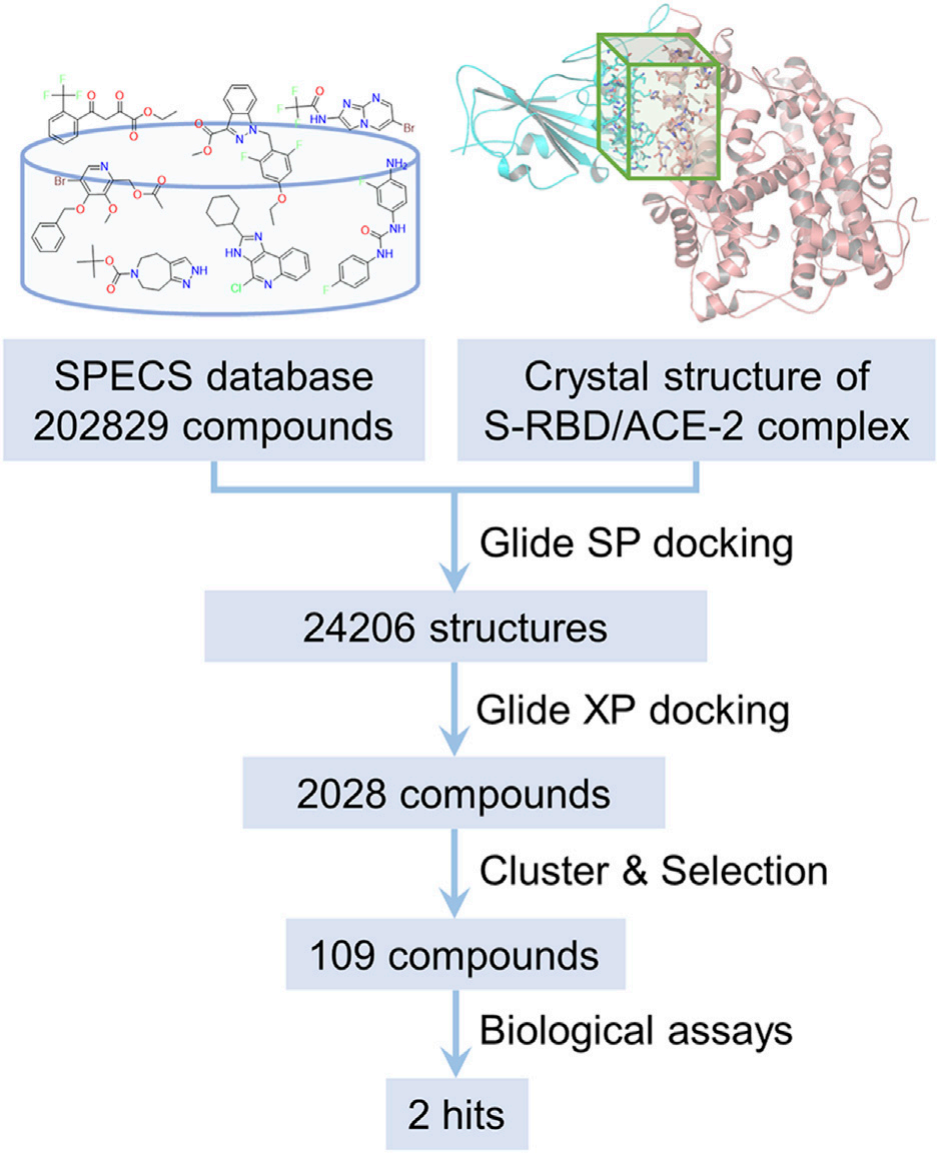
The flow chart of the discovery of inhibitors against SARS-CoV-2 S-RBD/ACE2 interaction by structure-based virtual screening.

In structure-based virtual screening, the primary thing is to choose the appropriate docking pocket and protein crystal structure. However, according to our observation, the SARS-CoV-2 S-RBD and human ACE2 don’t have druggable pockets near the contact interface in their unbound forms. Only when they are bound together, there is a well-defined pocket presented on their contact interface, which is hence defined as the docking pocket in the current study. A crystal structure of the SARS-CoV-2 S-RBD/human ACE2 complex was obtained from PDB and pretreated for virtual screening. The compounds for virtual screening were obtained from the SPECS database and first filtered by PAINS rules. The 202,829 remaining compounds were docking to the previously selected pocket after ligand preparation. The molecular docking was first carried out in standard precision mode. The top 10% poses ranked by SP score were redocked in extra precision mode. The top 20% candidates ranked by XP score were subsequently subjected to cluster analysis and visual inspection to pick the compounds with diversity and reasonable binding mode. Finally, 109 candidate compounds were selected and then purchased for follow-up biological testing. The whole process of the discovery of inhibitors against the interaction between S-RBD and ACE2 protein is schematically depicted in Figure 2.

### Identification of SARS-CoV-2 S-RBD/ACE2 Interaction Inhibitors Based on NanoBiT Assay

**FIGURE 3:**
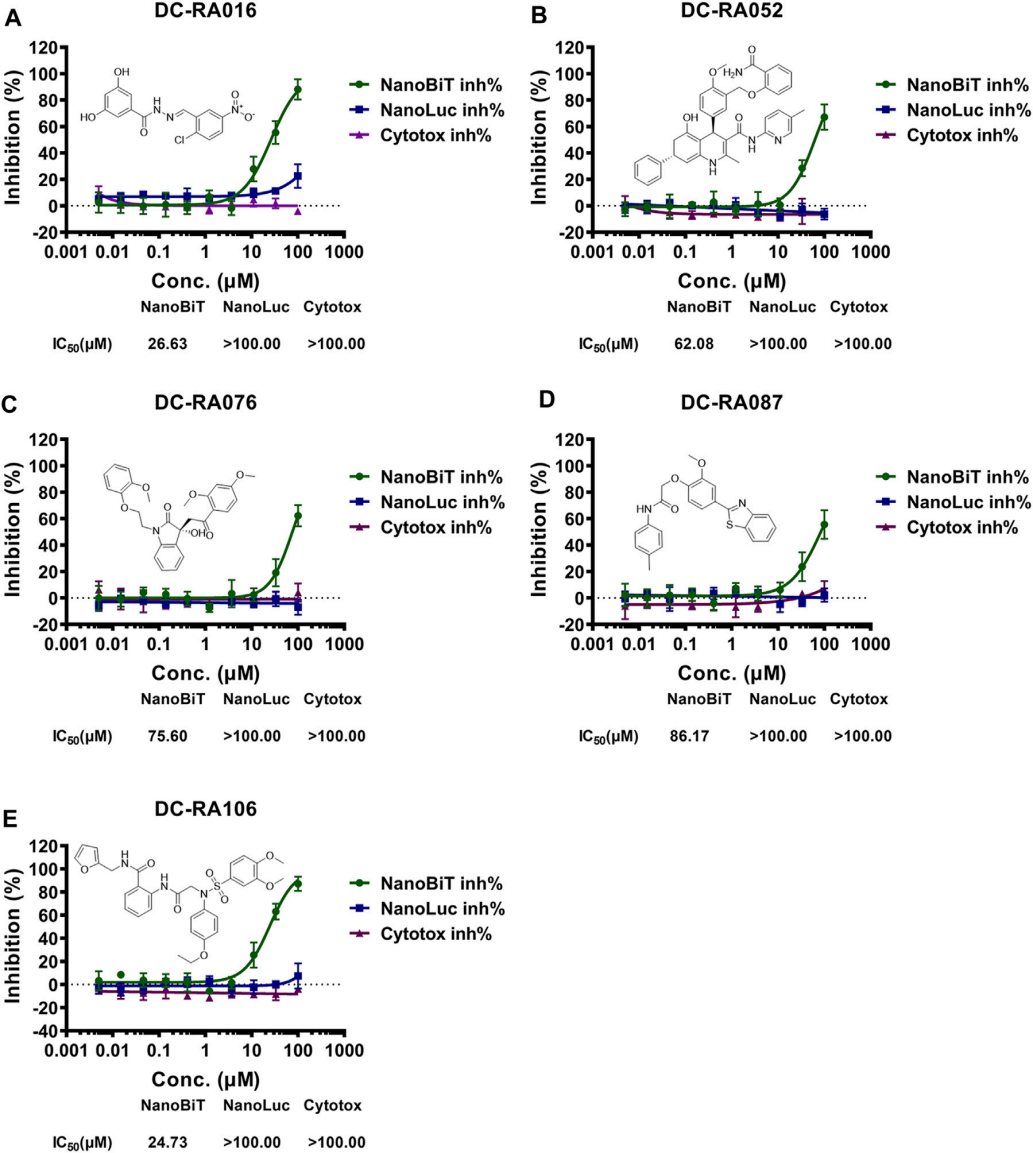
NanoBiT-based SARS-CoV-2 S-RBD/ACE2 interaction assays for five compounds: **(A)** DC-RA016; **(B)** DC-RA052; **(C)** DC-RA076; **(D)** DC-RA087; **(E)** DC-RA106. NanoBiT inh%: the inhibition rates against SARS-CoV-2 S-RBD/ACE2 interaction; NanoLuc inh%: the inhibition rates against NanoLuc luciferase; Cytotox inh%: the inhibition rates against the transfected HEK293 cell proliferation. *n* = 3.

**FIGURE 4:**
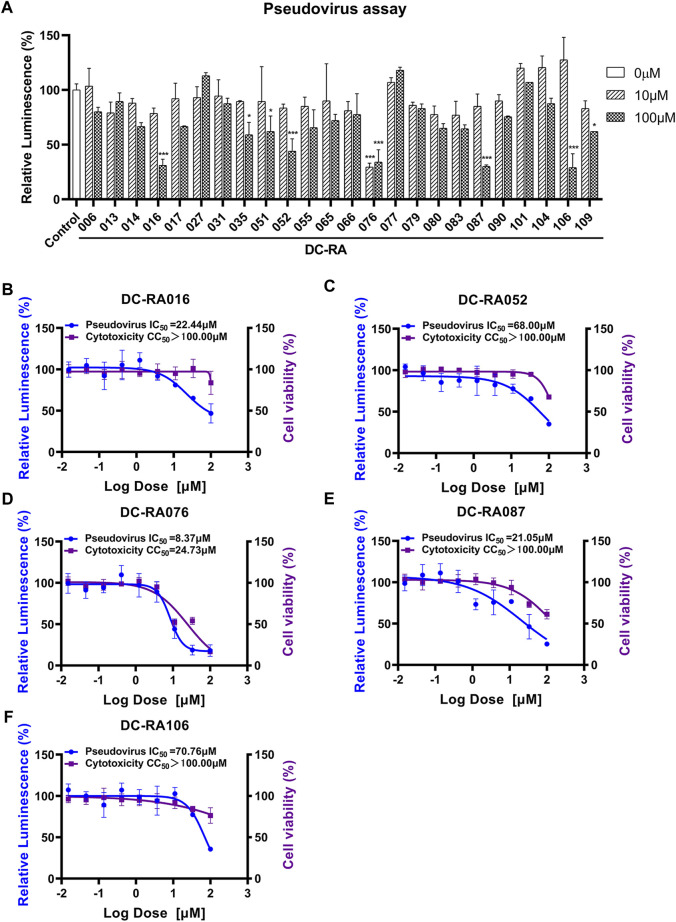
Anti-SARS-CoV-2-S pseudovirus activities and cytotoxicities of several compounds. **(A)** Determining Anti-SARS-CoV-2-S pseudovirus activities of 24 compounds showing the potent blocking activities against SARS-CoV-2 S-RBD/ACE2 interaction in the initial NanoBiT assay using the two concentrations (10, 100 μM); **(B-F)** IC_50_ and CC_50_ values of five compounds: **(B)** DC-RA016, **(C)** DC-RA052, **(D)** DC-RA076, **(E)** DC-RA087, **(F)** DC-RA106; Relative Luminescence (%) standing for the infection of SARS-CoV-2-S pseudovirus (blue), cell viability (purple).

To discover SARS-CoV-2 S-RBD/ACE2 interaction inhibitors, a NanoBiT-based assay was applied for preliminary screening. 24 of 109 compounds showed the primary inhibitory activities against SARS-CoV-2 S-RBD/ACE2 interaction under 20 and 50 μM concentration, no inhibitory effects on NanoLuc luciferase and cytotoxicity (Supplementary Figure S1). We next assessed these compounds for their antiviral activities against SARS-CoV-2-S pseudovirus. Among of them, five compounds were identified as potential inhibitors against SARS-CoV-2-S pseudovirus (Figure 4A). We further determined IC_50_ values of these compounds with serially-diluted concentrations for SARS-CoV-2 S-RBD/ACE2 interaction (NanoBiT inh%), NanoLuc luciferase (NanoLuc inh%) and CC_50_ values for the cytotoxicity (Cytotox inh%) on HEK293 cells (Figure 3). It was shown that DC-RA016, DC-RA052, DC-RA076, DC-RA087 and DC-RA106 exhibited dose-dependent inhibition against SARS-CoV-2 S-RBD/ACE2 interaction, and their IC_50_ values were 26.63, 62.08, 75.60, 86.17 and 24.73 μM, respectively. These compounds had no obvious inhibitory activities against NanoLuc luciferase (IC_50_ > 100 μM). It was observed that these compounds were low cytotoxicity with CC_50_ greater than 100 μM. Thus, the five compounds could disrupt the interaction between SARS-CoV-2 S-RBD and ACE2.

### Evaluation of Viral Attachment Inhibitors Using a SARS-CoV-2-S Pseudovirus-Based Inhibition Assay

To evaluate whether the compounds can inhibit the attachment of SARS-CoV-2, a pseudovirus based inhibition assay was established. Due to 24 compounds showing the potent blocking activities against SARS-CoV-2 S-RBD/ACE2 interaction in the initial NanoBiT assay, we next detected the inhibitory activities of these compounds in SARS-CoV-2-S pseudovirus based inhibition assay with the final concentrations of 10 and 100 μM. As shown in Figure 4A, five compounds significantly inhibited SARS-CoV-2-S pseudovirus attachment to ACE2-expressing HEK293T cells at a concentration of 100 μM (*p* < 0.001). Further experiments showed that DC-RA016, DC-RA052, DC-RA076, DC-RA087, and DC-RA106 exhibited dose-dependent inhibitory activities against pseudovirus attachment with IC_50_ values of 22.44, 68.00, 8.37, 21.05 and 70.76 μM, respectively (Figures 4B–F). Along with the pseudovirus assay, the cytotoxicity of the five compounds to ACE2-expressing HEK293T cells was also investigated (Figures 4B–F). It could be noted that the compound DC-RA076 showed obvious cytotoxicity (CC_50_ = 24.73 μM) to ACE2-expressing HEK293T cells with SI of 2.95 and MNCC of 2.64 μM (Table 1), while other four compounds displayed the inhibitory effects on SARS-CoV-2-S pseudovirus attachment without obvious cytotoxicity (CC_50_ > 100 μM) with SI ranging from 1.41 to 4.75 and MNCC ranging from 13.24 to 95.81 μM (Table 1). It is worth noting that the %I of DC-RA016 (52.08%), DC-RA052 (45.30%), and DC-RA087 (36.74%) is much larger than DC-RA076 (5.75%) and DC-RA106 (0.14%), indicating that the three compounds may have the potential for further structural optimization and fight against SARS-CoV-2. Thus, among the five compounds, DC-RA016, DC-RA052, and DC-RA087 demonstrated the relatively good antiviral effects with low cytotoxicity.

**TABLE 1:** Summary of Anti-SARS-CoV-2-S pseudovirus activities and cytotoxicities of five compounds.

Compounds	IC_50_ (μM)	CC_50_ (μM)	MNCC (μM)	%I	SI
DC-RA016	22.44	>100.00	95.81	52.08	>4.46
DC-RA052	68.00	>100.00	48.45	45.30	>1.47
DC-RA076	8.37	24.73	2.64	5.75	2.95
DC-RA087	21.05	>100.00	14.01	36.74	>4.75
DC-RA106	70.76	>100.00	13.24	0.14	>1.41

IC_50_: the 50% inhibitory concentration of Anti-SARS-CoV-2-S pseudovirus; CC_50_: cytotoxicities on ACE2-expressing HEK293T cells; MNCC: maximum non-cytotoxic concentration; %I: SARS-CoV-2-S pseudovirus inhibition ratio at MNCC; SI: selectivity index.

### Determination of Interactions Between the Five Compounds and SARS-CoV-2 S-RBD or ACE2 by SPR Assay

**FIGURE 5:**
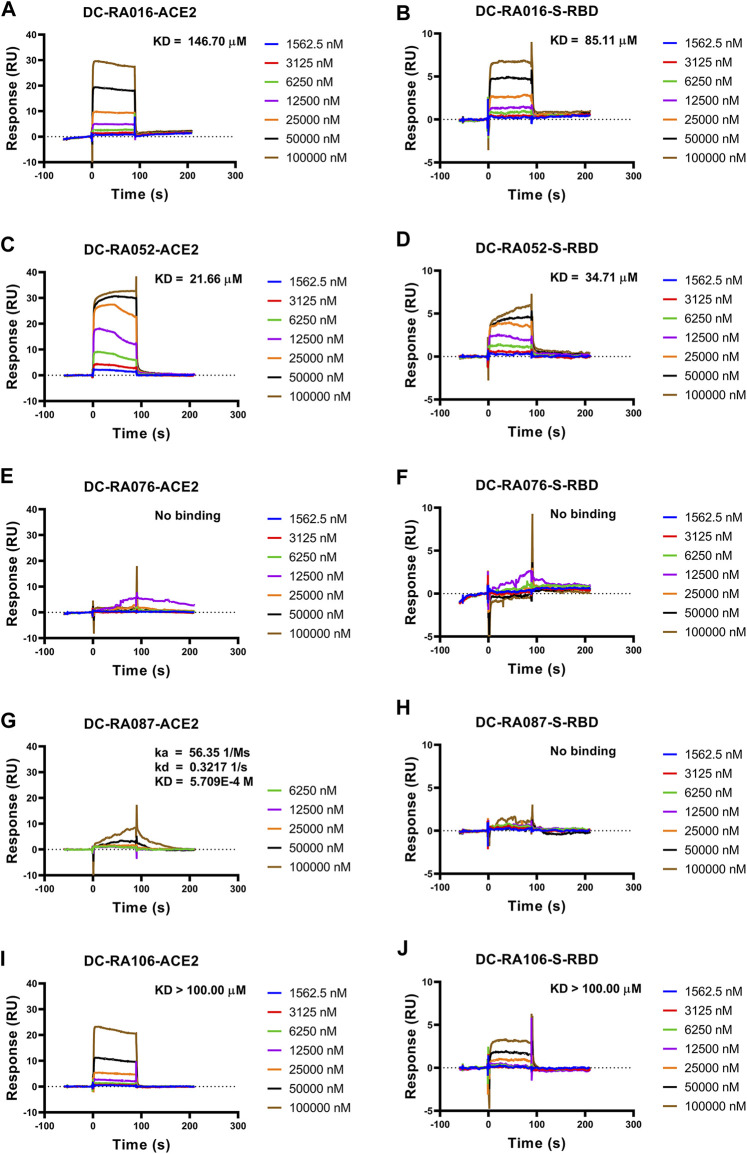
The binding affinity between the five compounds and SARS-CoV-2 S-RBD or ACE2. Compounds bound to SARS-CoV-2 S-RBD or ACE2. Interactions of SARS-CoV-2 S-RBD or ACE2 with compounds measured by SPR. The SARS-CoV-2 S-RBD or ACE2 was coated on the CM5 sensor chip and serial dilutions of compounds (typically, 1,562.5, 3,125, 6,250, 12,500, 25,000, 50,000 and 100,000 nM) were used as analytes. Changes in plasmon resonance are shown as response units. **(A)** Binding curves (colored lines) obtained by passing different concentrations of DC-RA016 over immobilized ACE2. **(B)** Binding curves (colored lines) obtained by passing different concentrations of DC-RA016 over immobilized S-RBD. **(C)** Binding curves (colored lines) obtained by passing different concentrations of DC-RA052 over immobilized ACE2. **(D)** Binding curves (colored lines) obtained by passing different concentrations of DC-RA052 over immobilized S-RBD. **(E)** Binding curves (colored lines) obtained by passing different concentrations of DC-RA076 over immobilized ACE2. **(F)** Binding curves (colored lines) obtained by passing different concentrations of DC-RA076 over immobilized S-RBD. **(G)** Binding curves (colored lines) obtained by passing different concentrations of DC-RA087 over immobilized ACE2. **(H)** Binding curves (colored lines) obtained by passing different concentrations of DC-RA087 over immobilized S-RBD. **(I)** Binding curves (colored lines) obtained by passing different concentrations of DC-RA106 over immobilized ACE2. **(J)** Binding curves (colored lines) obtained by passing different concentrations of DC-RA106 over immobilized S-RBD.

To further validate the inhibitory mechanism of the five compounds against SARS-CoV-2-S pseudovirus attachment, SPR assay was carried out to investigate whether these compounds could directly bind to SARS-CoV-2 S-RBD or ACE2. As shown in Figure 5, DC-RA016, DC-RA052, DC-RA087 and DC-RA106 could bind to human ACE2 (Figures 5A,C,G,I); while DC-RA016, DC-RA052 and DC-RA106 showed binding to SARS-CoV-2 S-RBD (Figures 5B,D,J). All these binding displayed fast kinetics, except for DC-RA087 to human ACE2 (Figure 5G). For DC-RA076, no binding was observed for both targets. DC-RA052 exhibited high affinities to both SARS-CoV-2 S-RBD and ACE2 and the KD values were 21.66 and 34.71 μM, respectively. In spite of concentration-dependent binding curves, saturated binding seemed not reached for DC-RA106, which could lead to poor quality of affinity regression (KD > 100.00 μM). In view of the previous results of the SARS-CoV-2-S pseudovirus-based inhibition assay, compounds DC-RA016 and DC-RA052 were ultimately identified as the two most promising hits blocking the interaction between SARS-CoV-2 S-RBD and ACE2 and SARS-CoV-2 virus attachment.

### Similarity Search and Structure-activity Relationship

Through the 2D similarity search, we found there were a few molecules similar to DC-RA016 in the SPECS database. Hence, 12 analogues of DC-RA016 were purchased to explore the structure-activity relationship of it. Their inhibition ability to the interaction of SARS-CoV-2 S-RBD and human ACE2 were tested by NanoBiT assay. As shown in Table 2, those compounds whose R1 groups were not 3,5-dihydroxy phenyl group all displayed no inhibitory activity against SARS-CoV-2 S-RBD and ACE2 interaction. Among the four compounds having the same R1 group with DC-RA016, two compounds (DC-RA016-9 and DC-RA016-12) could disrupt the interaction between SARS-CoV-2 S-RBD and ACE2 under the concentration of 50 and 20 μM. We further determined their dose-dependent inhibition against SARS-CoV-2 S-RBD/ACE2 interaction, NanoLuc luciferase and cytotoxicity on HEK293 cells. It was found that DC-RA016-12 exhibited the similar activity (IC_50_ = 26.06 μM) (Supplementary Figure S2A) to DC-RA016 (IC_50_ = 26.63 μM), while DC-RA016-9 displayed less inhibitory activity (IC_50_ = 73.84 μM) (Supplementary Figure S2B) than DC-RA016.

**TABLE 2:** The activities of DC-RA016 analogues in NanoBiT-based assay.

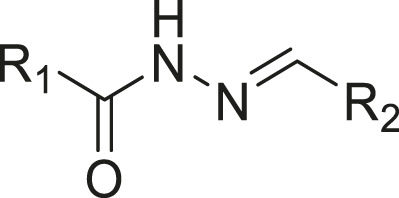
Cmpd name	R_1_	R_2_	SARS-CoV-2 S-RBD/ACE2 inhibition %		NanoLuc inhibition %	
			50 μM	20 μM	50 μM	20 μM
DC-RA016	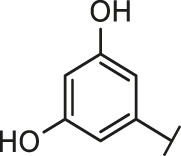	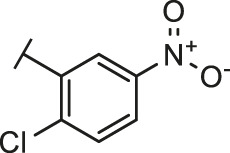	73.1	73.1	38.4	32.8
DC-RA016-1	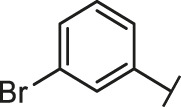	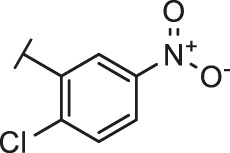	7.4	3.1	−2.2	0.6
DC-RA016-2	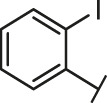	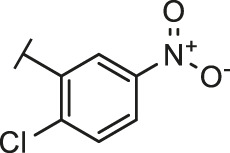	22.5	0.8	16.4	16.4
DC-RA016-3	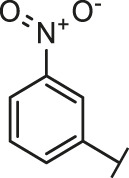	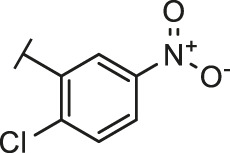	24.2	1.7	20.2	19.3
DC-RA016-4	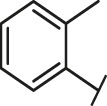	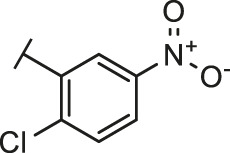	−24.9	−3.8	9.0	8.2
DC-RA016-5	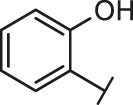	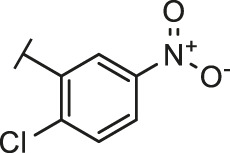	24.9	8.7	11.2	9.8
DC-RA016-6	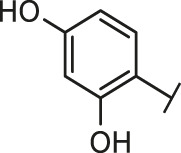	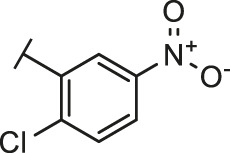	2.9	1.9	3.5	0.9
DC-RA016-7	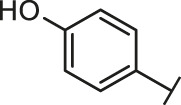	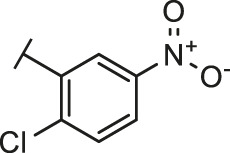	20.2	10.6	16.0	14.6
DC-RA016-8	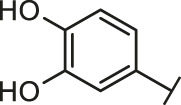	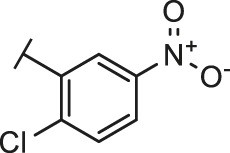	−18.3	6.7	5.7	0.0
DC-RA016-9	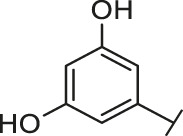	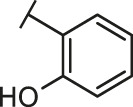	88.6	0.6	27.7	14.0
DC-RA016-10	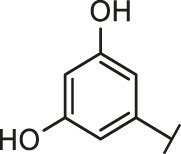	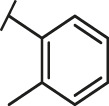	−17.7	−6.5	3.8	4.1
DC-RA016-11	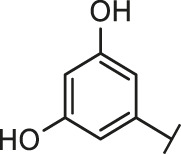	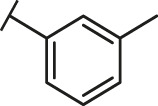	12.7	4.8	15.5	13.4
DC-RA016-12	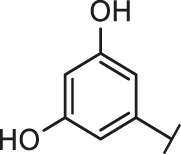	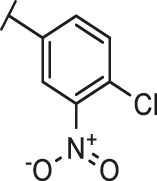	88.9	61.3	26.1	18.1

SARS-CoV-2 S-RBD/ACE2 Inhibition %: the inhibition rates against SARS-CoV-2 S-RBD/ACE2 interaction; NanoLuc Inhibition %: the inhibition rates against NanoLuc luciferase.

**FIGURE 6:**
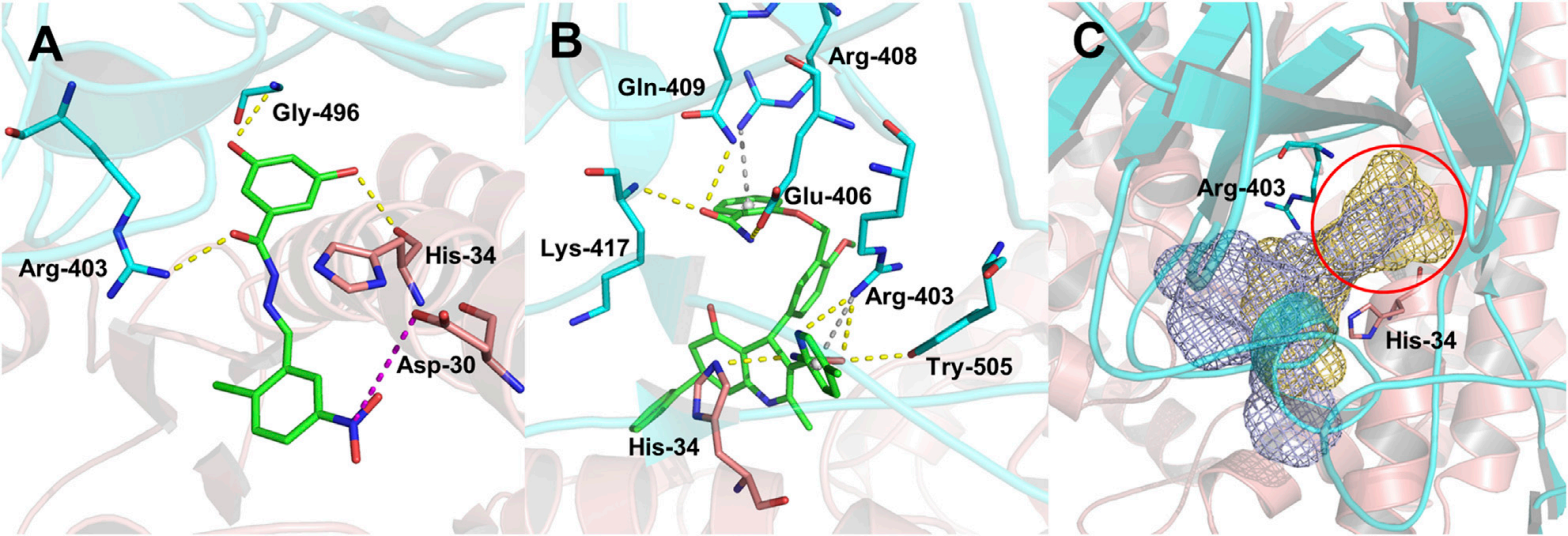
Analysis of binding modes of DC-RA016 and DC-RA052. **(A)** The lowest energy docked poses of DC-RA016 on the SARS-CoV-2 S-RBD/ACE2 interface. **(B)** The lowest energy docked poses of DC-RA052 on the SARS-CoV-2 S-RBD/ACE2 interface. **(C)** Superimposition of the docked poses of DC-RA016 and DC-RA052 (yellow and purple mesh). The green sticks denote ligands, the critical residues in binding cavities are shown as blue sticks (S-RBD) and pink sticks (ACE2), and the overall protein structures are shown as a blue cartoon (S-RBD) and a pink cartoon (ACE2).

To analyze the mechanism of action of DC-RA016, the binding pose of the compound DC-RA016 on the SARS-CoV-2 S-RBD/ACE2 interface was generated by XP docking. As shown in Figure 6A, DC-RA016 could form strong interactions with the SARS-CoV-2 S-RBD/ACE2 complex interface. The two phenolic hydroxyls of DC-RA016 respectively formed H-bonds with the His-34 of ACE2 and Gly496 of S-RBD. The replacement of the R1 group of DC-RA016 with other structures would disrupt such interaction, which provided the structural explanation for the impaired activity of compounds DC-RA016-1 to DC-RA016-8. Additionally, an electrostatic interaction also occurred between the nitro of DC-RA016 and Asp-30 of ACE2, which accounted for the stronger inhibitory activity of compounds DC-RA016 and DC-RA016-12 than compounds DC-RA016-9, DC-RA016-10, and DC-RA016-11. Compared with DC-RA016, the change of the position of chlorine substitution in DC-RA016-12 didn’t have obvious effects on its inhibitory activity. Moreover, although its R_2_ group was quite different from that of DC-RA016, DC-RA016-9 still displayed certain inhibitory activity. These results suggested that the R_2_ group was not the essential pharmacophore of DC-RA016, so we plan to carry further structural modification on this group in the future.

The docking analysis on DC-RA052, another important inhibitor, was also carried out. As shown in Figure 6B, Lys-417, Gln-409, Glu-406, Arg-403, Tyr-505 of SARS-CoV-2 S-RBD, and His-34 of ACE2 were found to form H-bonds with DC-RA052. Moreover, the benzene ring and pyridine ring of DC-RA052 formed π-cation interaction with the Arg-408 and Arg-403 of S-RBD. Comparing the binding modes of DC-RA016 and DC-RA052, it could be noted that both DC-RA016 and DC-RA052 interacted with the Arg-403 of S-RBD and His-34 of ACE2. Then, based on the docked complex structures, we calculated the per-residue interaction scores with glide. The results indicated the strong interaction of Arg-403 of S-RBD and His-34 of ACE2 to DC-RA016 and DC-RA052 (Supplementary Table S1). Moreover, as shown in Figure 6C, there was a deep pocket (marked with the red circle) on the SARS-CoV-2 S-RBD/ACE2 interface. Arg-403 of S-RBD and His-34 of ACE2 were both at the edge of this pocket. Hence, we considered Arg-403 of S-RBD and His-34 of ACE2 as two key residues responsible for the binding of ligands to the SARS-CoV-2 S-RBD/ACE2 interface. To explore the reason why the binding of identified inhibitors can influence SARS-CoV-2 S-RBD/ACE2 interaction, the mean effects of mutation of each residue within 5 Å of DC-RA016 and DC-RA052 on the binding affinity of SARS-CoV-2 S-RBD to ACE2 were visualized. The data for visualization was obtained from a previous study, where Starr et al. systematically changed every amino acid in the SARS-CoV-2 S-RBD and determined the effects of the substitutions on ACE2 binding. The mean effects per site were calculated from the set of Δlog_10_(K_D_, app) measurements of all missense mutations at a site (Starr et al., 2020). Δlog_10_(K_D_, app) represents the log binding constants relative to the wild-type SARS-CoV-2 RBD. As shown in Supplementary Figure S3, the mutations of many residues around DC-RA016 and DC-RA052 such as Ile-418 and Phe-497 could significantly reduce the binding affinity of SARS-CoV-2 S-RBD to ACE2, which mean these residues played key roles in the binding of SARS-CoV-2 S-RBD to ACE2. And yet, binding to the SARS-CoV-2 S-RBD/ACE2 interface, DC-RA016 and DC-RA052 would contact with these residues and interfere with their functions, disrupting the interaction between SARS-CoV-2 S-RBD and ACE2.

## Conclusion

Prior work has demonstrated that the interaction of SARS-CoV-2 S-RBD and the ACE2 receptor plays a critical role in the virus invasion into the host cell. Hence interference of SARS-CoV-2 S-RBD/ACE2 interaction is regarded as a promising antiviral strategy for SARS-CoV-2. However, only fewer small molecule inhibitors targeting such interaction have been reported so far. In this study, the structural-based virtual screening was conducted to search for the compounds that can inhibit the SARS-CoV-2 S-RBD/ACE2 interaction. The screened compounds were docked by targeting the SARS-CoV-2 S-RBD/ACE2 interface. Subsequently, A NanoBiT-based binding assay was performed to evaluate the inhibition effect of those compounds on SARS-CoV-2 S-RBD/ACE2 interaction resulting in 24 potential inhibitor candidates. Among them, DC-RA016, DC-RA052, and DC-RA087 displayed low cytotoxicity and moderate inhibition ability to SARS-CoV-2-S pseudovirus. Furthermore, the SPR assays identified that DC-RA016 and DC-RA052 could directly bond to both ACE2 and S-RBD. Taken together, the compounds DC-RA016 and DC-RA052 obtained in this study can serve as an ideal starting point for drug design against SARS-CoV-2 S-RBD/ACE2 interaction and SARS-CoV-2 infection. In addition, two biological active analogues of DC-RA016 were discovered through the 2D similarity search. Among them, DC-RA016-12 exhibited similar activity to DC-RA016 in NanoBiT-based assay.

## Data Availability

The original contributions presented in the study are included in the article/Supplementary Files, further inquiries can be directed to the corresponding authors.
