# Supplementary material for: Structure-Based Virtual Screening and Identification of Potential Inhibitors of SARS-CoV-2 S-RBD and ACE2 Interaction
Source: Front Chem. 2021 Sep 27;9:740702. doi: 10.3389/fchem.2021.740702 (PMC8504695; doi:10.3389/fchem.2021.740702)
Supplement: Supplementary file 1 [file Table1.docx]

Supplementary Material

for

**Structure-based virtual screening and identification of potential inhibitors of SARS-CoV-2 S-RBD and ACE2 interaction**

**Jiacheng Xiong^1,2†^, Yusen Xiang^3†^, Ziming Huang^1,2^, Xiaohong Liu^1,2,4^, Mengge Wang^3^, Guangbo Ge^3^, Hongzhuan Chen^3^, Jianrong Xu^5,6*^, Mingyue Zheng^1,2*^, Lili Chen^3*^**

^1^Drug Discovery and Design Center, State Key Laboratory of Drug Research, Shanghai Institute of Materia Medica, Chinese Academy of Sciences, 555 Zuchongzhi Road, Shanghai 201203, China

^2^College of Pharmacy, University of Chinese Academy of Sciences, No. 19A Yuquan Road, Beijing 100049, China

^3^Institute of Interdisciplinary Integrative Medicine Research, Shanghai University of Traditional Chinese Medicine, 1200 Cailun Road, Shanghai 201203, China

^4^Shanghai Institute for Advanced Immunochemical Studies, School of Life Science and Technology, ShanghaiTech University, 393 Middle Huaxia Road, Shanghai 200031, China

^5^Academy of Integrative Medicine, Shanghai University of Traditional Chinese Medicine, 1200 Cailun Road, Shanghai 201203, China

^6^Department of Pharmacology and Chemical Biology, Shanghai Jiao Tong University School of Medicine, 280 South Chongqing Road, Shanghai 200025, China

**
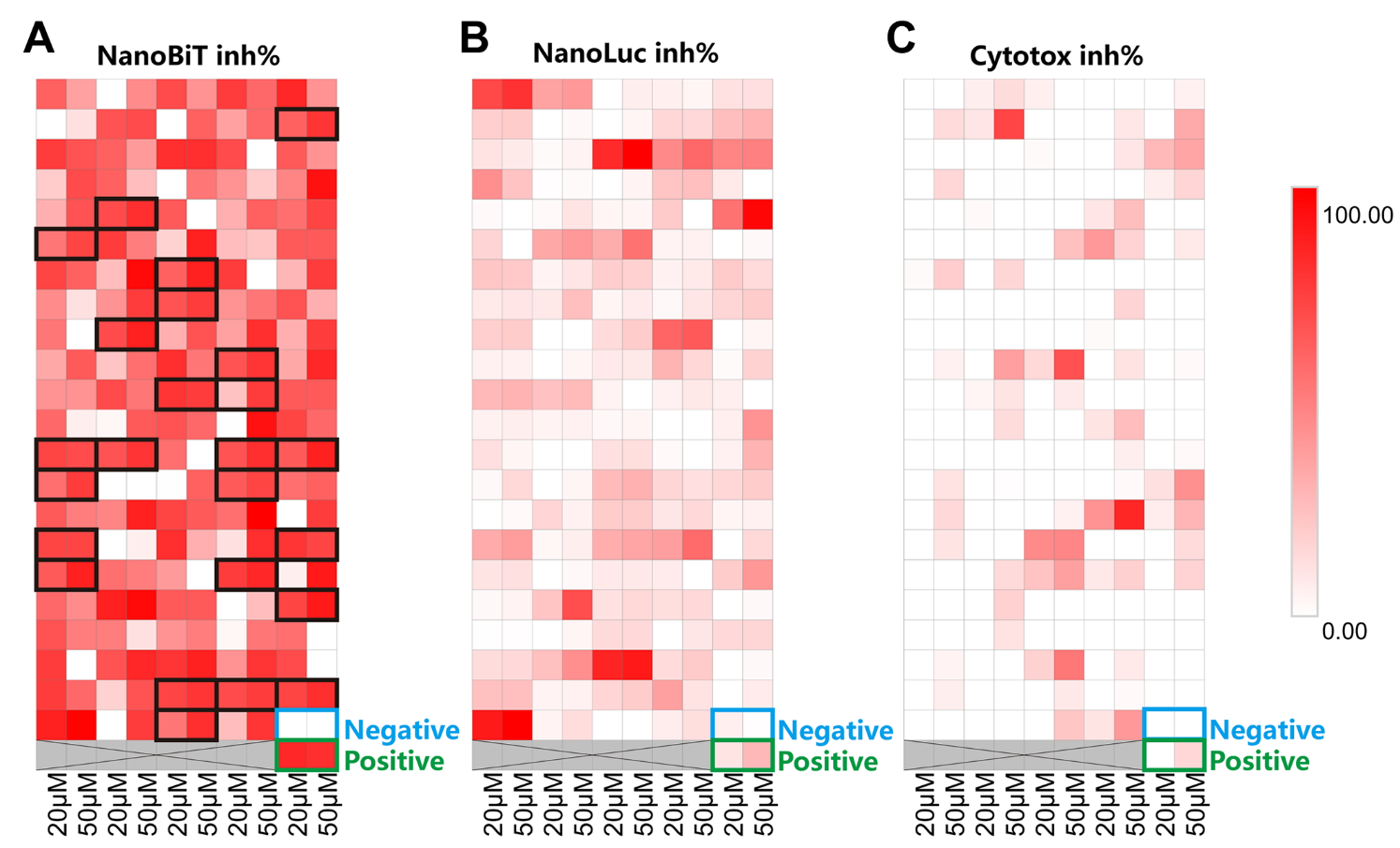
**

**Figure S1.** NanoBiT-based screening to identify the active compounds that block the interaction between SARS-CoV-2 S-RBD and ACE. Heatmap illustration of effects of 109 compounds at low (20 μM) and high (50 μM) concentrations on SARS-CoV-2 S-RBD/ACE2 interaction **(A)**, Nanoluc luciferase **(B)** and cell proliferation **(C)**. Positive (glycyrrhizic acid) and negative (ginsenoside Rb1) controls are shown which have been reported in our previous publication (Yu et al., 2020). The colors indicate the inhibitory activities, and color intensity represents potency. The figures were generated by Morpheus (https://software.broadinstitute.org/morpheus).

**
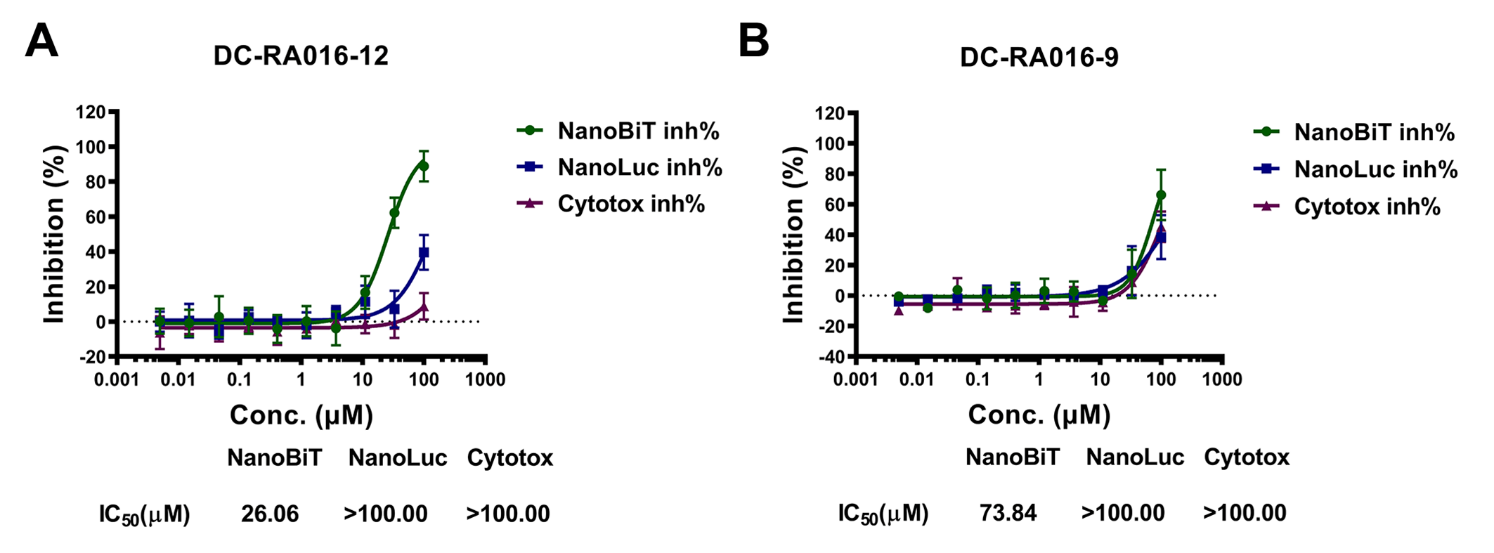
Figure S2.** NanoBiT-based SARS-CoV-2 S-RBD/ACE2 interaction assays for DC-RA016 analogues: DC-RA016-12 **(A)** and DC-RA016-9 **(B)**. NanoBiT inh%: the inhibition rates against SARS-CoV-2 S-RBD/ACE2 interaction; NanoLuc inh%: the inhibition rates against NanoLuc luciferase; Cytotox inh%: the inhibition rates against the transfected HEK293 cell proliferation. *n*=3.

**Table S1.** The per-residue interaction scores of residues interacting with DC-RA016 and DC-RA052

| Ligand | Residues | Per-residue interaction scores |
| --- | --- | --- |
| DC-RA016 | **His-34 (ACE2)** | -10.010 |
|  | **Arg-403 (S-RDB)** | -7.519 |
|  | Gly-496 (S-RDB) | -3.591 |
|  | ASP-30 (ACE2) | -0.438 |
| DC-RA052 | **Arg-403 (S-RDB)** | -32.370 |
|  | Lys-417 (S-RDB) | -30.360 |
|  | Arg-408 (S-RDB) | -25.639 |
|  | **His-34 (ACE2)** | -8.349 |
|  | Try-505 (S-RDB) | -2.532 |
|  | Gln-409 (S-RDB) | -0.148 |
|  | Glu-406 (S-RDB) | 2.721 |

**
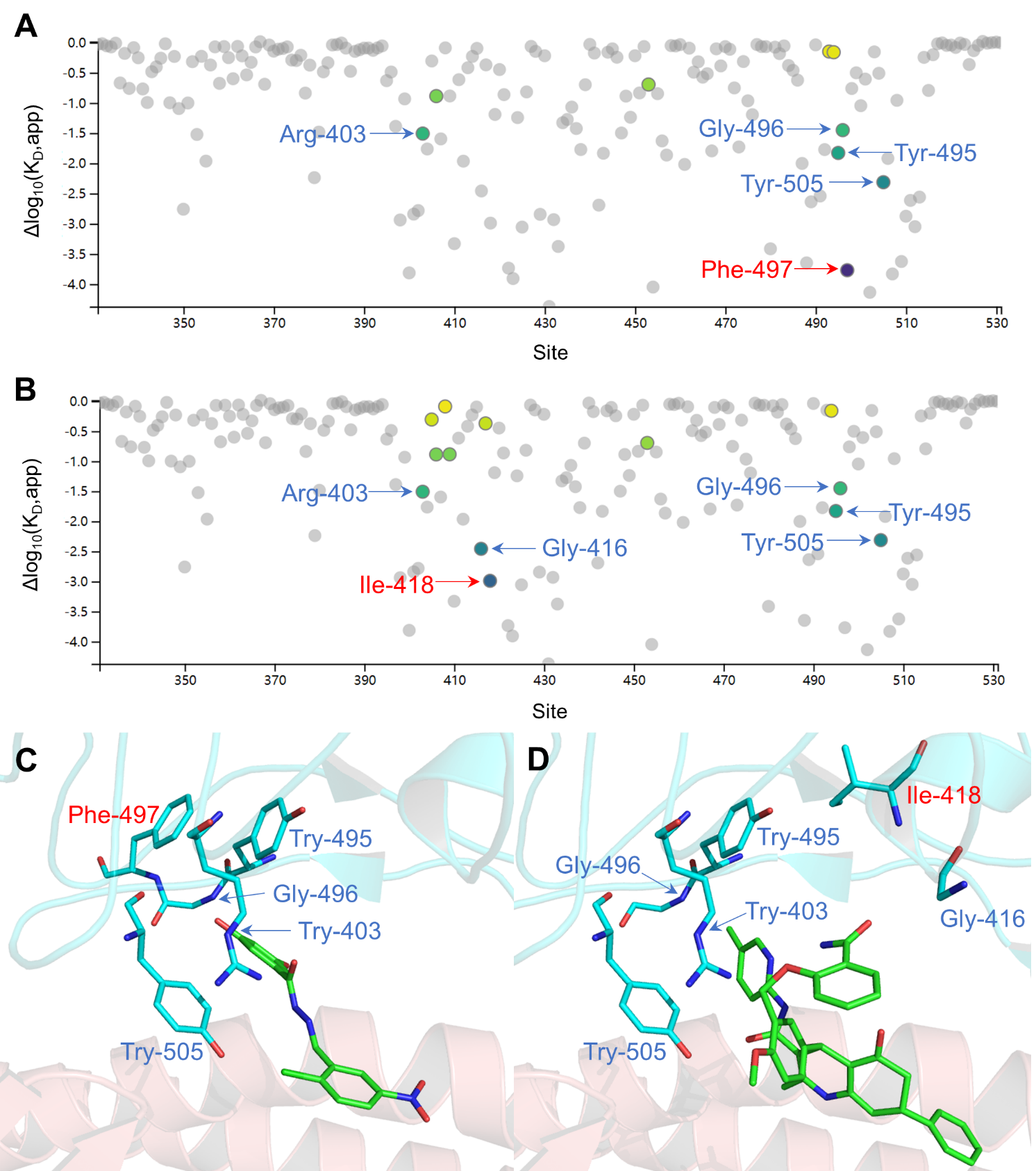
**

**Figure S3.** Analysis of the effects of ligand binding on SARS-CoV-2 S-RBD/ACE2 interaction. **(A-B)** The mean effects of mutations on each residue around DC-RA016 **(A)** and DC-RA052 **(B)** on the binding affinity of SARS-CoV-2 S-RBD to ACE2. The mean effects per site are calculated from the set of Δlog_10_(K_D_, app) measurements of all missense mutations at a site. The Δlog_10_(K_D_, app) represents the log binding constants relative to the wild-type SARS-CoV-2 RBD. The residues within 5 Å of DC-RA016 and DC-RA052 are colored by the value of Δlog_10_(K_D_, app). The figures were generated at https://jbloomlab.github.io/SARS-CoV-2-RBD_DMS/structures/. **(C-D)** Some key residues around DC-RA016 **(C)** and DC-RA052 **(D)** that are important for the binding of SARS-CoV-2 S-RBD to ACE2. The overall protein structures are shown as a blue cartoon (S-RBD) and a pink cartoon (ACE2).
